# Association of cigarette smoking habits with the risk of prostate cancer: a systematic review and meta-analysis

**DOI:** 10.1186/s12889-023-16085-w

**Published:** 2023-06-15

**Authors:** Xiangwei Yang, Hong Chen, Shiqiang Zhang, Xianju Chen, Yiyu Sheng, Jun Pang

**Affiliations:** 1 Department of Urology, Kidney and Urology Center, Pelvic Floor Disorders Center, The Seventh Affiliated Hospital, Sun Yat-Sen University, No.628 Zhenyuan Road, Shenzhen, 518107 China; 2 School of Nursing, LKS Faculty of Medicine, University of Hong Kong, Hong Kong, China

**Keywords:** Cigarette smoking, Prostate cancer, Risk, Systematic review, Meta-analysis

## Abstract

**Background:**

Association of cigarette smoking habits with the risk of prostate cancer is still a matter of debate. This systematic review and meta-analysis aimed to assess the association between cigarette smoking and prostate cancer risk.

**Methods:**

We conducted a systematic search on PubMed, Embase, Cochrane Library, and Web of Science without language or time restrictions on June 11, 2022. Literature search and study screening were performed according to the Preferred Reporting Items for Systematic Reviews and Meta-Analyses statement. Prospective cohort studies that assessed the association between cigarette smoking habits and the risk of prostate cancer were included. Quality assessment was conducted using the Newcastle–Ottawa Scale. We used random-effects models to obtain pooled estimates and the corresponding 95% confidence intervals.

**Results:**

A total of 7296 publications were screened, of which 44 cohort studies were identified for qualitative analysis; 39 articles comprising 3 296 398 participants and 130 924 cases were selected for further meta-analysis. Current smoking had a significantly reduced risk of prostate cancer (RR, 0.74; 95% CI, 0.68–0.80; *P* < 0.001), especially in studies completed in the prostate-specific antigen screening era. Compared to former smokers, current smokers had a significant lower risk of PCa (RR, 0.70; 95% CI, 0.65–0.75; *P* < 0.001). Ever smoking showed no association with prostate cancer risk in overall analyses (RR, 0.96; 95% CI, 0.93–1.00; *P* = 0.074), but an increased risk of prostate cancer in the pre-prostate-specific antigen screening era (RR, 1.05; 95% CI, 1.00–1.10; *P* = 0.046) and a lower risk of prostate cancer in the prostate-specific antigen screening era (RR, 0.95; 95% CI, 0.91–0.99; *P* = 0.011) were observed. Former smoking did not show any association with the risk of prostate cancer.

**Conclusions:**

The findings suggest that the lower risk of prostate cancer in smokers can probably be attributed to their poor adherence to cancer screening and the occurrence of deadly smoking-related diseases, and we should take measures to help smokers to be more compliant with early cancer screening and to quit smoking.

**Trial registration:**

This study was registered on PROSPERO (CRD42022326464).

**Supplementary Information:**

The online version contains supplementary material available at 10.1186/s12889-023-16085-w.

## Background

Prostate cancer (PCa) is the second most commonly diagnosed cancer and the fifth leading cause of cancer death among males, with an estimated 1.4 million new cases and 375 000 deaths worldwide in 2020, accounting for 7.3% and 3.8% of all cancers diagnosed, respectively [1]. Various endogenous and exogenous risk factors for PCa have been discussed for decades. Several factors have been identified to be associated with an increased risk of PCa, for instance, family history [2], elevated hormone levels [2], black ethnicity [2], and high alcohol consumption [3]. Conversely, several factors have been associated with a decreased risk of PCa, such as higher intake of tomatoes [4], increased coffee consumption [5] and sexual activity [6].

Smoking is a well-established risk factor for several cancers, such as lung cancer, head and neck cancer, bladder cancer, and esophageal cancer [7, 8]. However, the data on the association between smoking and PCa incidence are conflicting [9, 10]. In a meta-analysis of 24 prospective cohort studies [11], M. Huncharek showed that current smokers had no increased risk of incident PCa, but in data stratified by amount smoked, a significant elevated risk was observed, and former smokers had a higher risk of PCa in comparison with never smokers. Another meta-analysis conducted in 2014 [12] revealed an inverse association between current smoking and PCa risk, while in studies completed before the prostate-specific antigen (PSA) screening era, ever smoking was positively associated with PCa. In addition, a recent pooled study of five Swedish cohorts [13] demonstrated that former smokers and current smokers had a lower risk of PCa than never smokers, and smoking intensity was inversely associated with PCa risk, especially in the PSA screening era.

Biological mechanisms underlying smoking and PCa risk have been studied for many years. Burning cigarettes can produce more than 7000 chemicals, and at least 70 carcinogens such as polycyclic aromatic hydrocarbons (PAHs) and cadmium [14]. Mutations or functional polymorphism in genes involved in PAH metabolism and detoxification may increase the risk of PCa [15]. The glutathione-S-transferases (GSTs) are a class of enzymes that can detoxify PAHs. The most common subtypes of GSTs in human prostate are GSTP and GSTM, which were reported to be associated with an increased risk of PCa in smokers [15, 16]. Cadmium induces prostate carcinogenesis through interaction with the androgen receptor because of its androgen-like activity, and it also enhances androgen-mediated transcriptional activity when in combination with the androgen [17]. A higher level of androgen was related to increased PCa risk [2, 18]. Smoking can increase testosterone concentrations by promoting testosterone secretion from Leydig cells or acting as an aromatase inhibitor [19]. Mutations in the p53 gene and CYP1A1 gene showed a higher risk of PCa in smokers, suggesting that smoking may have a joint effect on PCa risk when combined with susceptible genotypes [20]. Increased heme oxygenase 1 (HO-1) messenger RNA expression and upregulated HO-1 protein levels were observed in PCa cell lines DU 145 and PC3 [21], implying that HO-1 may play a role in the development of PCa for its function in promoting angiogenesis [22]. Evidence also suggested that prostatic inflammation may be involved in the development and progression of PCa [23]. Cigarette smoke augments the production of numerous pro-inflammatory cytokines, decreases the levels of anti-inflammatory cytokines, and activates macrophage and dendritic cell activity in many ways [24].

We performed this systematic review and meta-analysis to investigate the association of cigarette smoking habits with the risk of PCa. We aimed to include a larger sample of studies than previous meta-analyses and collect the latest evidence and the most comprehensive information on the association between cigarette smoking and PCa risk. Our primary objective was to assess the risk of PCa in current smokers, former smokers, and ever smokers. We hypothesized that smokers have a higher risk of PCa compared to non-smokers.

## Methods

### Search strategy

This systematic review and meta-analysis was performed according to the Preferred Reporting Items for Systematic Reviews and Meta-Analyses (PRISMA) statement [25]. Two independent investigators (XWY and HC) searched PubMed, Embase, Cochrane Library, and Web of Science for publications from database inception to June 11, 2022. The following search terms were used: ("Prostate cancer") AND ("Cigarette" OR "Smoking" OR "Tobacco") AND ("Risk" OR "Incidence"). No language restrictions were applied. Reference lists of identified articles and relevant reviews were screened for additional studies. Details of the protocol for this systematic review were registered on PROSPERO and can be accessed by CRD42022326464.

### Selection criteria

Prospective cohort studies investigating the association between cigarette smoking and PCa risk were included for analysis. The primary outcome was the risk of PCa. Those studies that provided an effect measure (i.e., a relative risk) quantifying the impact of smoking on the risk of PCa were considered for further quantitative synthesis (meta-analysis). The removal of duplicates and assessment of article eligibility were conducted independently by XWY and HC, and any disagreements were resolved by consulting the senior author (JP). Review articles, editorials, meeting abstracts, case‒control studies, cross-sectional studies, and those not on the topic were excluded.

### Quality assessment

All included studies were independently assessed by XWY and HC for risk of bias using the Newcastle‒Ottawa Scale for cohort studies [26]. This scale assesses the selection of the study groups, the comparability of the groups, and the ascertainment of the outcome of interest. Studies with 7–9 scores were considered to be of high quality, those with 5–6 scores were classified as intermediate quality, and those with less than 4 scores were classified as low quality. Disagreements in the quality assessment were resolved by consulting JP.

### Data extraction

Data were independently extracted by XWY and HC. All extracted variables were cross-checked to ensure their reliability. We recorded the total number of participants, PCa cases, and the mean or median follow-up time across all included studies. Relative risks (RRs) and the corresponding 95% confidence intervals (CIs) were retrieved or calculated using frequency distributions. Considering the prevalence rate of PCa in the public, we believed that the odds ratio was close to the RR [27, 28]. Hazard ratios (HRs) and RRs are different, HRs contain temporal information but RRs do not [28]. We converted HRs to RRs based on the formula provided by Shor E et al. [29], and the corresponding 95% CIs were converted using the same method. RRs and 95% CIs of ever smokers were computed by combining the results for former and current smokers when these results were not reported in the original papers. In addition, we recorded the baseline characteristics, methods, adjusted confounding factors, and other important comments to establish comparability. Discrepancies were discussed and resolved by consensus.

### Statistical analysis

Three authors (SZQ, XJC and YYS) performed statistical analyses using Stata software, version 16.0 (StataCorp). When both crude and adjusted RRs were provided, we used the most fully adjusted value. We calculated the pooled RRs and 95% CIs and plotted forest plots using random-effects models (DerSimonian‒Laird method) for the association of current smoking, former smoking, and ever smoking with the risk of PCa [30]. Statistical heterogeneity across the trials was assessed using the I^2^ statistic and the Cochran’s Q test. Values of the I^2^ statistic of approximately 25%, 50%, and 75% were interpreted as low, moderate, and high heterogeneity, respectively [31]. In the case of low heterogeneity, a fixed-effects model (Inverse variance method) was applied. We plotted funnel plots and used Egger’s test to examine publication bias. Additionally, a series of sensitivity analyses were performed to assess the robustness of our results. We stratified studies by reference status (never smoker, former smokers), completion year (pre-PSA screening era vs. PSA screening era), world region (North America vs. Europe vs. Asia vs. Australia), and the Newcastle‒Ottawa Scale score (≤ 6 points vs. > 6 points). We considered 1995 as a cutoff year of study completion to distinguish studies before and after the PSA screening era [12]. All tests were two-tailed, and *P* < 0.05 was considered statistically significant.

## Results

### Study population

**Fig. 1:**
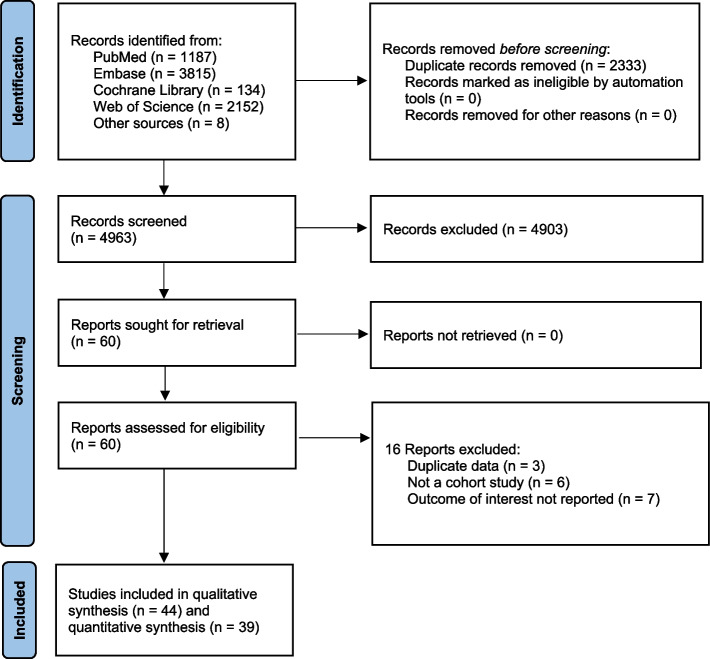
Flow diagram of included studies

We identified 7296 citations, and after removing duplicates, 4963 citations remained for screening. After the removal of ineligible citations, we retained 60 articles that we assessed for eligibility by reading the full text; 16 of these were excluded for specific reasons. Finally, 44 studies met our inclusion criteria for qualitative synthesis and meta-analysis (Fig. 1). The number of participants and PCa cases from each selected study for systematic review ranged separately from 997 to 844 455 and 54 to 40 821, with a median of 22 677 and 382, respectively. Overall, 39 studies with 3 296 398 participants and 130 924 cases were identified for meta-analysis, and 5 studies with 91 377 participants and 1364 cases were not included in meta-analysis due to lack of information (Additional file 1). Articles were published between 1989 and 2022 and were from studies conducted in the following geographic regions: 19 from Europe (4 from the United Kingdom, 4 from Norway, 3 from Sweden, 2 from Finland, 1 from France, 1 from the Netherlands, 1 from Denmark, 1 from Lithuania, 1 from Iceland, and one from 10 European countries), 18 from North America (17 from the United States, 1 from Canada), 5 from Asia (3 from Japan, 1 from South Korea, 1 from Singapore), and 2 from Australia. The median score of quality assessment for all eligible studies was 7, with a range of 6–9 (Additional file 2).

### Current smoking

**Fig. 2:**
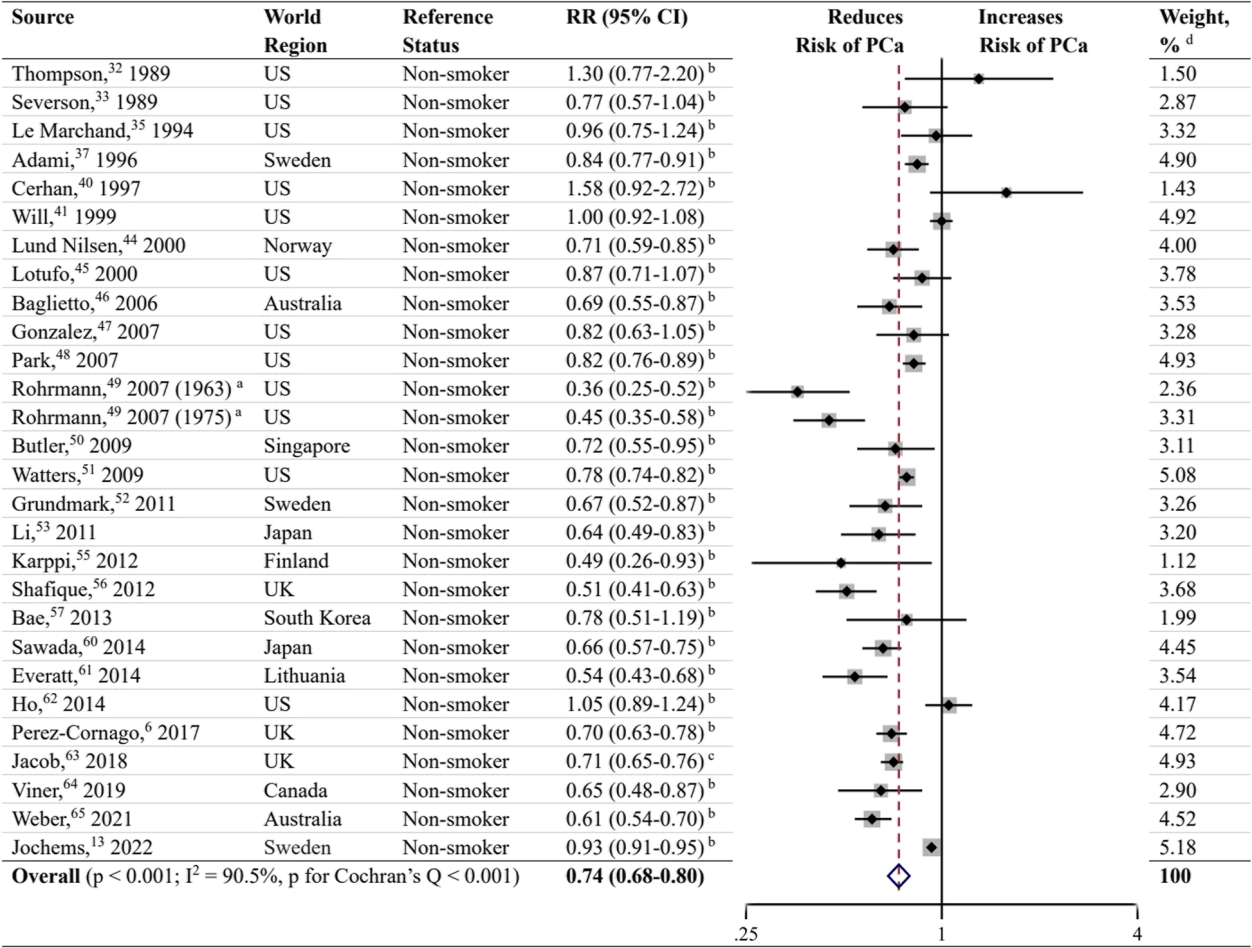
Forest plot for the association between current smoking and prostate cancer. RR, relative risk; CI, confidence interval; PCa, prostate cancer; US, United States; UK, United Kingdom. ^a^ Rohrmann et al. [49] had two sub-populations. ^b^ RR and 95% CI were calculated using frequency distributions. ^c^ RR and 95% CI were converted from HR and corresponding 95% CI using the formula RR ≈ (1-e ^HR x ln (1−P0)^)/P_0_ (P_0_ refers to the incidence rate of PCa in the control group). ^d^ Weights were from random effects analysis

**Fig. 3:**
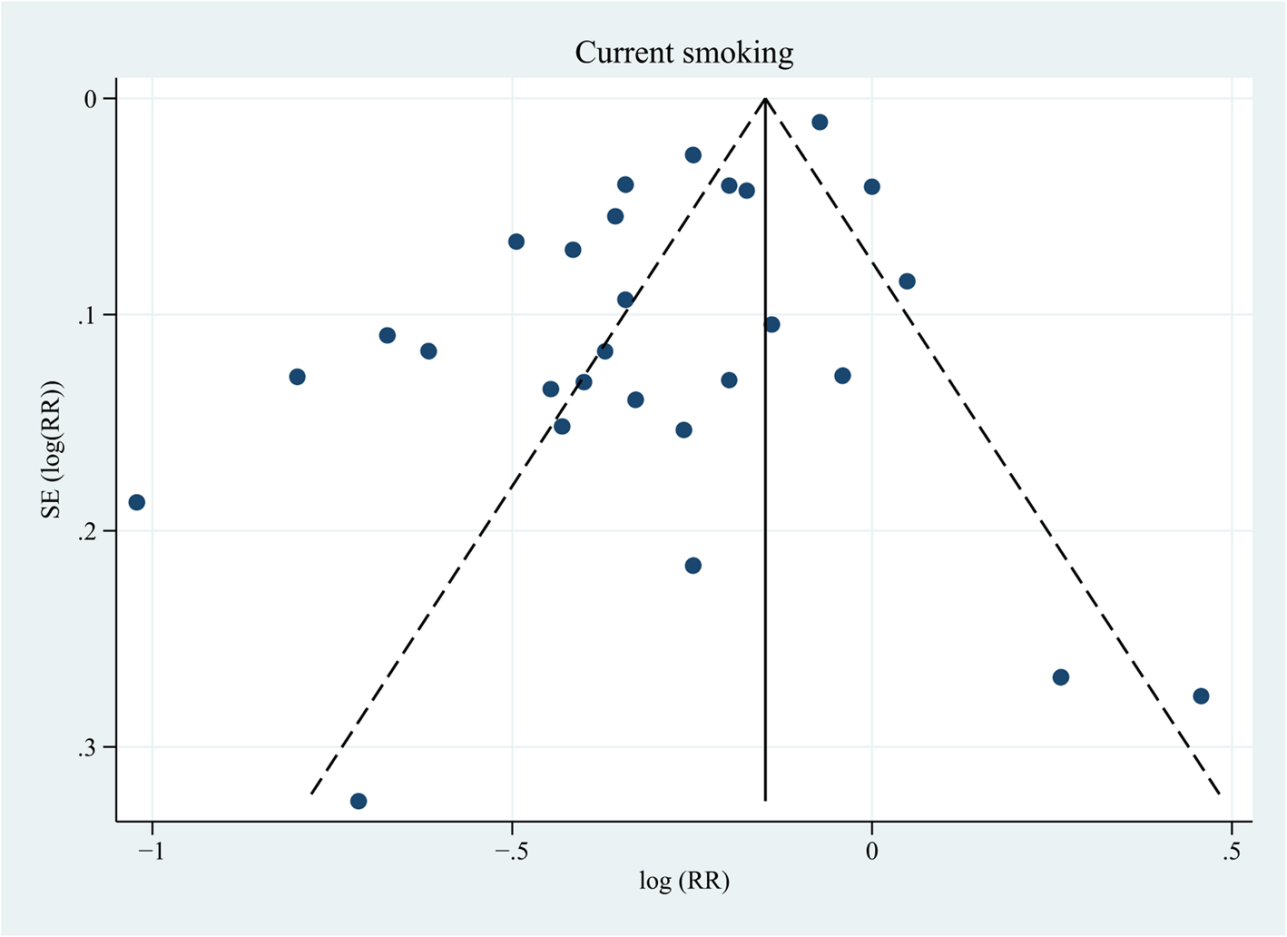
Funnel plot for publication bias in the studies investigating current smoking and prostate cancer risk. SE, standard error. Twenty-eight dots from 27 studies. *P* = 0.231

In total, 37 studies [6, 13, 32–66] reported the risk of current smoking on PCa, among which 6 studies [32, 35, 41, 53, 55, 63] took non-smokers as the reference and the remaining 31 studies [6, 13, 33, 34, 36–40, 42–52, 54, 56–62, 64–66] took never smokers as the reference. We defined non-smokers as never smokers plus former smokers. RRs and 95% CIs of current smokers versus non-smokers were calculated using frequency distributions in never smokers and former smokers when the risk estimates were not provided in original studies. Ten studies [34, 36, 38, 39, 42, 43, 54, 58, 59, 66] did not provide enough data on frequency distribution and were not included in analysis. Twenty-seven studies [6, 13, 32, 33, 35, 37, 40, 41, 44–53, 55–57, 60–65] were included to calculate the pooled RR and 95% CI. The results showed that current smoking at baseline was associated with a reduced risk of PCa (RR, 0.74; 95% CI, 0.68–0.80; *P* < 0.001) (Fig. 2). The I^2^ statistic and the Cochran’s Q test showed high heterogeneity (I^2^ = 90.5%; *P* < 0.001). Inspection of the funnel plot did not demonstrate publication bias (*P* = 0.231; Fig. 3).

When performing sensitivity analyses (Additional file 3) stratified by reference status, studies using never smokers as the reference [6, 13, 33, 34, 36–40, 42–52, 54, 56–62, 64–66] showed a similar inverse association with PCa risk (RR, 0.90; 95% CI, 0.86–0.95; *P* < 0.001), with the heterogeneity lower than that of analysis of studies using non-smokers as the reference (I^2^ = 66.7%; *P* < 0.001). Compared to former smokers, current smokers had a significant lower risk of PCa (RR, 0.70; 95% CI, 0.65–0.75; *P* < 0.001) based on 21 studies [6, 13, 33, 37, 40, 44–52, 56, 57, 60–62, 64, 65]. In the pre-PSA screening era, current smoking showed a decreased risk of PCa (RR, 0.79; 95% CI, 0.64–0.98; P = 0.033) compared to non-smokers, while in the PSA screening era, the risk was significantly lower (RR, 0.72; 95% CI, 0.66–0.79; *P* < 0.001). When stratified by world region, studies conducted in North America, Europe, Asia, and Australia showed a negative association between current smoking and PCa risk. We also performed subgroup analyses in 21 studies with quality scores ≥ 7 [6, 13, 32, 33, 35, 40, 44–46, 49–52, 55–57, 60–62, 64, 65] and 6 studies with quality scores of 6 [37, 41, 47, 48, 53, 63]. Thereupon, both demonstrated a reduced risk of PCa.

### Former smoking

**Fig. 4:**
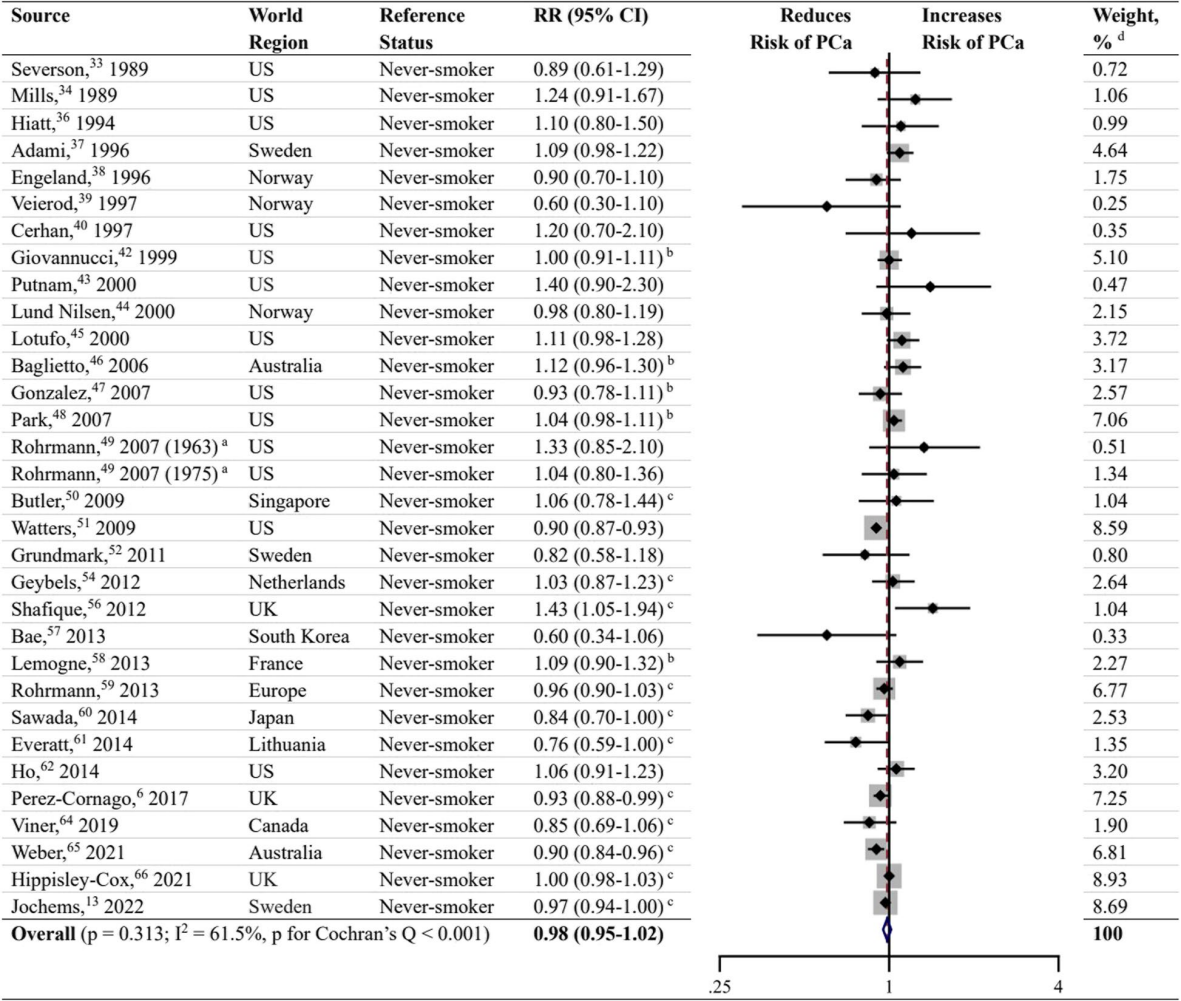
Forest plot for the association between former smoking and prostate cancer. ^a^ Rohrmann et al. [49] had two sub-populations. ^b^ RR and 95% CI were calculated using frequency distributions. ^c^ RR and 95% CI were converted from HR and corresponding 95% CI using the formula RR ≈ (1-e ^HR x ln (1−P0)^)/P_0_ (P_0_ refers to the incidence rate of PCa in the control group). ^d^ Weights were from random effects analysis

**Fig. 5:**
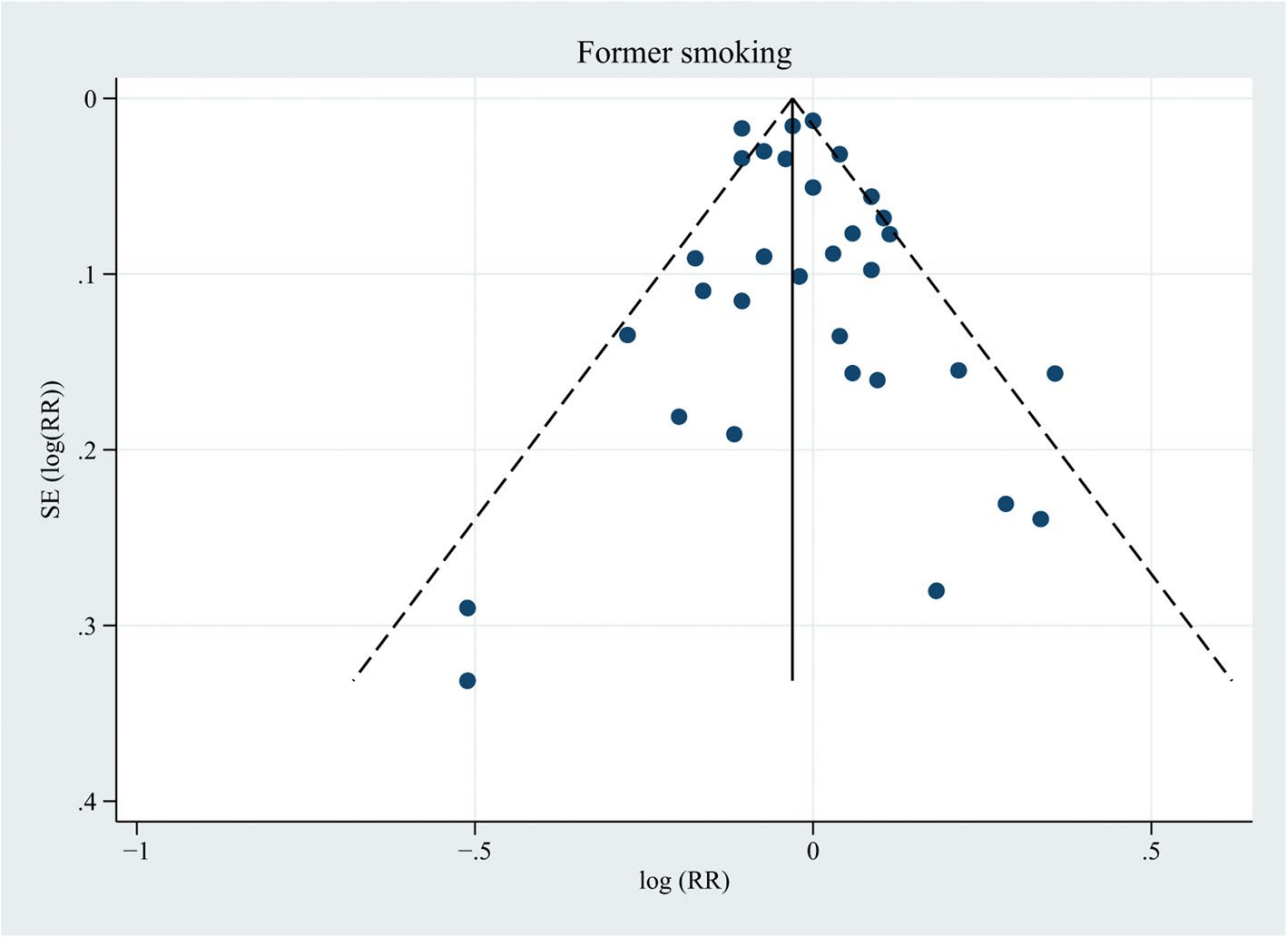
Funnel plot for publication bias in the studies investigating former smoking and prostate cancer risk. Thirty-two dots from 31 studies. *P* = 0.431

Meta-analysis on former smoking as a risk factor for PCa was performed in 31 studies (Fig. 4) [6, 13, 33, 34, 36–40, 42–52, 54, 56–62, 64–66], and the results showed no significant association between former smoking and the risk of PCa (RR, 0.98; 95% CI, 0.95–1.02; *P* = 0.313). The data were heterogeneous according to the I^2^ statistic and the Cochran’s Q test (I^2^ = 61.5%; *P* < 0.001). Inspection of the corresponding funnel plot did not show evidence of publication bias (*P* = 0.431; Fig. 5). Sensitivity analyses stratified by PSA screening era, world region, and quality score also demonstrated no association between former smoking and PCa risk (Additional file 3).

### Ever smoking

**Fig. 6:**
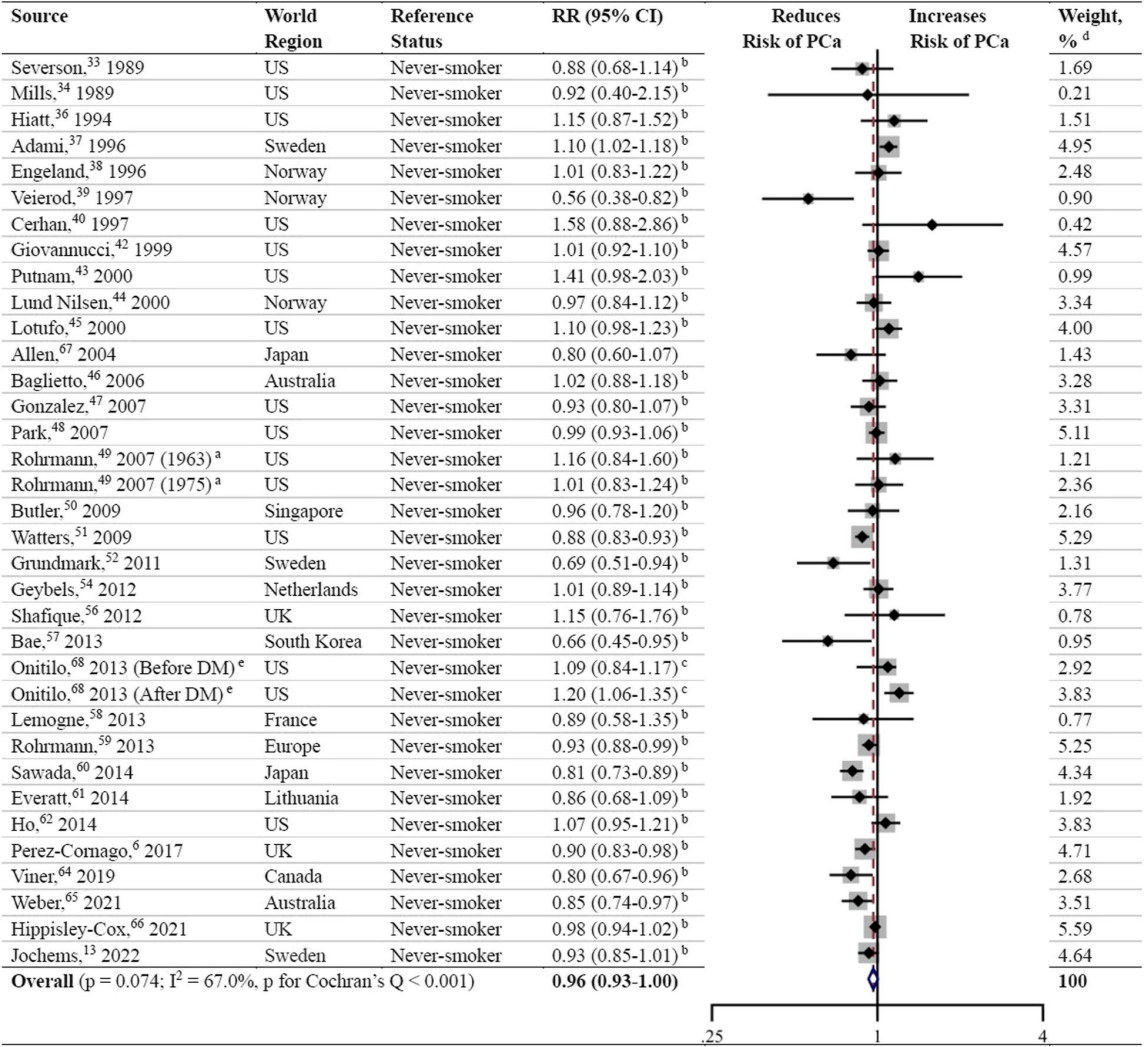
Forest plot for the association between ever smoking and prostate cancer. DM, diabetes mellitus. ^a^ Rohrmann et al. [49] had two sub-populations. ^b^ RR and 95% CI were calculated using frequency distributions or risk estimates and 95% CIs in subgroups. ^c^ RR and 95% CI were converted from HR and corresponding 95% CI using the formula RR ≈ (1-e ^HR x ln (1−P0)^)/P_0_ (P_0_ refers to the incidence rate of PCa in the control group). ^d^ Weights were from random effects analysis. ^e^ Onitilo et al. [68] had two sub-populations

**Fig. 7:**
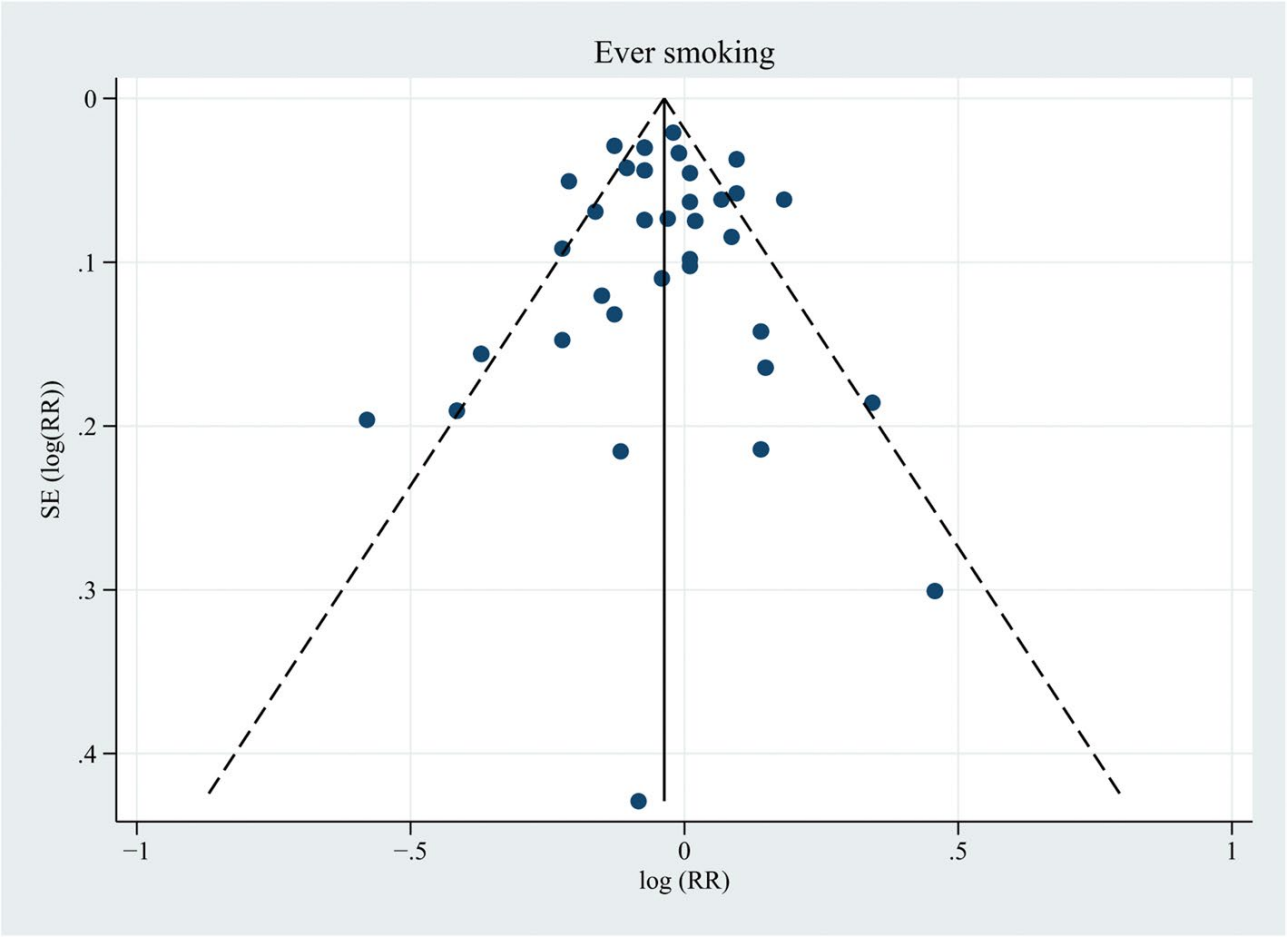
Funnel plot for publication bias in the studies investigating ever smoking and prostate cancer risk. Thirty-five dots from 33 studies. *P* = 0.672

Thirty-three studies were included in the meta-analysis to assess the association of ever smoking with the risk of PCa (Fig. 6) [6, 13, 33, 34, 36–40, 42–52, 54, 56–62, 64–68]. Two of those studies [67, 68] provided RRs and 95% CIs in the original paper, and the risk estimates of the remaining 31 studies [6, 13, 33, 34, 36–40, 42–52, 54, 56–62, 64–66] were calculated by combining results for former and current smokers. Thereupon, the pooled RR and 95% CI showed no association with the risk of PCa (RR, 0.96; 95% CI, 0.93–1.00; *P* = 0.074), with an I^2^ value of 67.0% and a negative result of publication bias ((*P* = 0.672; Fig. 7). The association was inverse when analyzing studies completed in the PSA screening era (RR, 0.95; 95% CI, 0.91–0.99; *P* = 0.011), but in the pre-PSA screening era, ever smokers showed a significantly increased risk of PCa compared to never smokers (RR, 1.05; 95% CI, 1.00–1.10; *P* = 0.046) (Additional file 3). Four studies [50, 57, 60, 67] from Asia showed a pooled reduced risk of PCa in ever smokers (RR, 0.82; 95% CI, 0.74–0.91; *P* < 0.001), and studies from North America, Europe, and Australia revealed no association between ever smoking and PCa incidence. In terms of subgroup analyses stratified by quality score, the studies with a quality score ≥ 7 showed a modest negative association with PCa risk (RR, 0.96; 95% CI, 0.92–1.00; *P* = 0.047), while the studies with a quality score of 6 showed no association.

### Studies not included in the meta-analysis

Of these 5 studies (Additional file 1) [69–73], 4 studies (involving 211 cases, 524 cases, 127 cases, and 129 cases) [69, 70, 72, 73] reported no significant association between cigarette smoking and the risk of PCa, 2 of which had a smoking category increment of 10 cigarette per day [69] or cigarette pack-years per 10 years [72]. The study conducted by Karlsen et al. [73] did not differentiate cigarette, cigar, cheroot, and pipe when assessing the risk of PCa in smokers, and as a result, this study could not be included in the meta-analysis. In the study conducted by Chamie et al. [71], a reduced PCa risk was reported in participants with a smoking history (with 13,144 participants and 363 cases; RR, 0.78; 95% CI, 0.72–0.85; *P* < 0.001).

## Discussion

In this systematic review and meta-analysis, we found that current smoking was inversely associated with the risk of PCa, especially in the PSA screening era, which was inconsistent with our hypothesis but was consistent with the results of the recent studies [12, 13]. In studies using never smokers as the reference, current smoking revealed a similar negative correlation with PCa risk, accompanied by less heterogeneity. Current smokers had a lower risk of PCa compared to former smokers. Former smoking and ever smoking were not associated with PCa risk in the overall analyses. However, when stratified by completion year, ever smoking showed an increased risk of PCa in the pre-PSA screening era and a lower risk of PCa in the PSA screening era. Studies from North America, Europe, Asia, and Australia showed a similar reduced PCa risk in current smokers compared to non-smokers, whereas in ever smokers, only studies conducted in Asia demonstrated a decreased risk of PCa. There are several explanations for these results. Current smoking was believed to be associated with a lower likelihood of PSA testing [74, 75], and individuals with a smoking history were less likely to undergo prostate biopsy [62, 76]. As a consequence, the detection rate of PCa could be relatively lower among participants in the PSA screening era. The difference in the patterns of the association between ever smoking and PCa risk in Asia and other regions can be attributed to the higher proportion of studies in the PSA screening era in Asia than afterward. Additionally, the differences in race/ethnicity, socioeconomic status, educational attainment, and health literacy may also play important roles in explaining regional distinctions [77–79]. In a national cross-sectional survey, PSA testing was significantly higher in US-born men and older non-Hispanic White men than in foreign-born men and men from other racial categories [77]. Another study revealed that White men aged > 50 years were more likely than Black men to undergo PSA testing, and those with lower socioeconomic status were associated with less PSA testing [78]. The association of education levels with the preference for PSA screening was inconsistent [77, 79]. Johnson JA et al. [77] declared that higher educational levels were associated with higher odds of ever having had a PSA test; however, Pickles K et al. [79] announced that the preference for PSA screening was stronger in those without tertiary education and with inadequate health literacy. The age of the participants in the selected studies varied widely, and therefore, the willingness to receive PSA screening differs considerably; older people often show poorer adherence to PSA testing guidelines [77]. On the other hand, the relationship between PSA levels and smoking is still a matter of debate. According to an Italian cross-sectional study [80], PSA accuracy was reported to be lower in smokers than in nonsmokers and former smokers, suggesting that the need for PSA-based prostate biopsy can be affected to a certain extent by smoking.

Another possible explanation is that smoking is the leading risk factor for death among males [81]. Smokers may die from smoking attributable diseases including cancers, cardiovascular diseases, and respiratory diseases before their diagnosis of PCa. The majority of cases of lung cancer [7], head and neck cancer [82], approximately 50% of bladder cancer cases [83], and 49% of esophageal squamous cell carcinoma cases [84] are caused by cigarette smoking. Furthermore, smoking was reported to cause nearly 90% of lung cancer deaths [7] and showed significant associations with poor survival in patients with head and neck cancer [85]. Moreover, the detection of asymptomatic PCa can be frequently ignored when focusing on a more aggressive cancer. In addition, smoking increases the risk for stroke and coronary heart disease by 2 to 4 times, and stroke and coronary heart disease are considered to be the leading causes of death in the United States [8], and most of these deaths are caused by smoking [86]. Smoking can also cause chronic obstructive pulmonary disease (COPD), increasing 12 to 13-fold risk of dying from COPD than nonsmokers [8], and nearly 80% of deaths from COPD can be ascribed to smoking [86].

Our study found an increased risk of PCa among ever smokers in the pre-PSA screening era, indicating that it is necessary to promote smoking cessation as early as possible. Nearly one in five deaths are caused by cigarette smoking in the United States, leading to more than 480 000 deaths each year [8]. Continued tobacco use has been shown to limit the effectiveness of major cancer treatments, increase the risk of treatment-related complications and the development of secondary cancers, and lower cancer survival rates and the quality of life of patients [7]. In patients with PCa, smokers at the time of PCa diagnosis are associated with more aggressive characteristics, and the risk of experiencing biochemical recurrence, distant metastasis, cancer-specific mortality, and overall mortality is much higher [9, 10, 12, 87, 88]. Nicotine-induced chronic prostatic inflammation [23, 89], aberrant CpG methylations of adenomatous polyposis coli and glutathione S-transferase pi are the potential biological mechanisms responsible for these [90]. Although the effect of smoking cessation on PCa progression remains unclear, the negative impact of smoking has suggested to be maintained as long as 10 years after smoking cessation [10]. Additionally, active smoking is associated with adverse reproductive health outcomes, type 2 diabetes mellitus, and rheumatoid arthritis, harming nearly every organ of the body and resulting in significant economic costs for smokers, their families, and society [7].

Much progress has been made in promoting smoking cessation in recent decades. However, it is far from sufficient. In 2018, 13.7% of all adults (34.2 million people) in the United States were reported as current cigarette smokers [91]. Of them, 55.1% had made an attempt to quit in the past 12 months, but only 7.5% achieved success. Overcoming both physical nicotine dependence and long-standing rewarding behavior is a huge challenge, and most individuals relapse within 3 months after quitting smoking [92]. Evidence has indicated that the combination of behavioral and pharmacological interventions produces the largest cessation effects [7, 8, 92]. Nevertheless, fewer than one-half of tobacco users were offered cessation treatment according to a survey of oncology providers [93], and the inability to get patients to quit and patient resistance to treatment are two dominant barriers to cessation intervention. A brief intervention may be more acceptable and sustainable to help smokers quit smoking, according to a randomized clinical trial performed at emergency departments in Hong Kong [94]. Quitlines are good alternatives to interventions for both patients and clinicians because of their convenience and specialization, and their roles in improving smoking cessation rates have been confirmed [95]. For smokers with time constraints, internet-based self-help materials such as the website smokefred.gov and newer smartphone applications have also shown benefits in promoting smoking cessation and can serve as good alternatives [96, 97].

### Strengths and limitations

The key strength of this systematic review is that the study comprised a total of 44 prospective cohort studies, 39 of which were included in the meta-analysis, with the largest number of participants and PCa cases to date. Furthermore, we included all the data on current smoking, former smoking, and ever smoking in the analysis without date and language restrictions, which means that the study provides the latest evidence and the most comprehensive information on the association between cigarette smoking and risk of PCa. We assessed the quality of each selected study using the Newcastle‒Ottawa Scale for cohort studies, and the median score was 7 and the lowest score was 6, suggesting that the quality of the included studies can be guaranteed. Other strengths include applying independent literature search, quality assessment, and data extraction by two investigators; conducting several sensitivity analyses; and using Egger’s test to examine publication bias.There are some limitations of our study. Most of the information on smoking habits was obtained from self-administered questionnaires, and the definitions of current smokers and former smokers were not completely the same between different studies. Some participants may have changed their smoking habits after baseline investigations, but repeated assessment of smoking exposure was absent in primary studies. We calculated RRs and the corresponding 95% CIs using frequency distributions without adjusting confounding factors when risk estimates were not reported. We focused on the impact of cigarette smoking on the risk of PCa; second-hand cigarette smoke and the use of other tobacco products (cigars, smokeless tobacco, e-cigarettes, pipes, etc.) that have showed increased risk of many cancers in numerous studies [98, 99] were not discussed. Alcohol consumption showed a significant dose–response relationship with PCa risk in several studies [3, 100], and were often used concurrently with cigarette smoking [101], but we didn’t analyze the effect of concurrent use of cigarette and alcohol on risk of PCa due to lack of information on alcohol consumption in the included studies. High heterogeneity was showed by the I^2^ statistics and the Cochran’s Q test, and the difference in adjusted confounding factors may be one of the reasons. We have included multivariate results as much as possible to reduce the bias, and there was no indication of publication bias. Dividing studies into pre-PSA screening era and PSA screening era based on publication year (1995 as the cut-off) may produce bias because many of the cohorts published and categorized into the PSA screening era extended into the pre-PSA screening era. Another limitation is that we failed to calculate the impact of quantitative cigarette consumption on the PCa risk due to a lack of data. However, we have to point out that the meta-regression conducted by Islami et al. [12] was methodologically wrong as including multiple data points from a single study with the same control group counts the effect of that control group multiple times (i.e., unit-of-analysis error).

## Conclusions

To the best of our knowledge, this systematic review and meta-analysis contained the largest sample of prospective cohort studies, the latest evidence and the most comprehensive information on the association between cigarette smoking habits and the risk of PCa. The smokers’ poor adherence to cancer screening and the occurrence of smoking-related aggressive cancers as well as cardiovascular, pulmonary, and several other deadly diseases may explain the negative association. Regional distinctions can be attributed to the difference of participants in age, ethnicity, socioeconomic status, and educational levels. In addition, a correct methodology is important, the choice of different effect models should base on the heterogeneity and characteristics of enrolled studies. However, it is difficult to conclude a positive association between cigarette smoking and PCa risk as we hypothesized due to these affecting factors. We should focus on taking measures to help smokers to be more compliant with early cancer screening and to quit smoking.

## Supplementary Information

**Additional file 1.** A. Characteristics of the 39 studies included in the meta-analysis. B. Characteristics of the 5 studies not included in the meta-analysis due to lack of information.

**Additional file 2.** Results of quality assessment using the Newcastle-Ottawa Scale for cohort studies.

**Additional file 3.** Sensitivity analyses of association between smoking status and risk of prostate cancer.

## Data Availability

All data generated or analyzed during this study are included in this published article and its supplementary information files.

## Abbreviations

PCa: Prostate cancer
PSA: Prostate-specific antigen
PAHs: Polycyclic aromatic hydrocarbons
GSTs: Glutathione-S-transferases
HO-1: Heme oxygenase 1
PRISMA: Preferred Reporting Items for Systematic Reviews and Meta-Analyses
Cig/d: Cigarettes per day
Pk-yr: Pack-year
Yr: Year
CI: Confidence interval
RR: Relative risk
FU: Follow-up
NR: Not reported
US: United States
BP: Blood pressure
BMI: Body mass index
UK: United Kingdom
DM: Diabetes mellitus
NS: Non-significant
COPD: Chronic obstructive pulmonary disease
