# Supplementary material for: Association of cigarette smoking habits with the risk of prostate cancer: a systematic review and meta-analysis
Source: BMC Public Health. 2023 Jun 15;23:1150. doi: 10.1186/s12889-023-16085-w (PMC10268475; doi:10.1186/s12889-023-16085-w)
Supplement: Supplementary file 1 — Additional file 1. A. Characteristics of the 39 studies included in the meta-analysis. B. Characteristics of the 5 studies not included in the meta-analysis due to lack of information. [file 12889_2023_16085_MOESM1_ESM.docx]

**Additional file 1A. Characteristics of the 39 studies included in the meta-analysis**

| **First author, publication year** | **Study name (or description); country, recruitment period** | **Study design, outcome** | **Last FU (FU^a^, yrs)** | **Total no. men/cases** | **Smoking category*** | **No. cases*** | **RR (95% CI) *** | **Variables the results were adjusted for; Effect measures in the original studies; Other comments** |
| --- | --- | --- | --- | --- | --- | --- | --- | --- |
| Thompson,^32^  1989 | Rancho Bernardo, California; US, 1972-1974 | Cohort, incidence | 1987 (NR) | 1776/ 54 | Non-smoker  Current | 43  11 | Referent  1.30 (0.77-2.20) ^b^ | Age, diabetes, heart disease, BP, plasma cholesterol, BMI, whole milk intake, egg intake;  RR; 90% CIs were converted to 95% CIs |
| Severson,^33^  1989 | Men of Japanese ancestry in Hawaii; US, 1965-1968 | Cohort, incidence | 1986 (NR) | 8006/174 | Never-smoker  Current  Former  Ever | 63  65  46  111 | Referent  0.87 (0.61-1.23)  0.89 (0.61-1.29)  0.88 (0.68-1.14) ^b^ | Age; RR |
| Mills,^34^  1989 | Adventist Health Study; US, 1976 | Cohort, incidence | 1982 (NR) | 35000/172 | Never-smoker  Current  Former  Ever | 90  3  79  82 | Referent  0.49 (0.16-1.57)  1.24 (0.91-1.67)  0.92 (0.40-2.15) ^b^ | Age; RR |
| Le Marchand,^35^  1994 | Hawaii State Department of Health cohort; US, 1975-1980 | Cohort, incidence | 1989 (NR) | 20316/198 | Non-smoker  Current | NR  NR | Referent  0.96 (0.75-1.24) ^b^ | Age, ethnicity, income; RR |
| Hiatt,^36^  1994 | Kaiser Permanente Medical Care Program; US, 1978-1985 | Cohort, incidence | 1985 (4.6) | 43432/238 | Never-smoker  Current  Former  Ever | 79  49  94  143 | Referent  1.38 (0.74-2.59) ^b^  1.10 (0.80-1.50)  1.15 (0.87-1.52) ^b^ | Age, alcohol consumption, smoking status, race, and education; RR |
| Adami,^37^  1996 | Swedish Construction workers; Sweden, 1971-1975 | Cohort, incidence | 1991 (18) | 135006/  2368 | Never-smoker  Current  Former  Ever | 682  1069  617  1686 | Referent  1.11 (1.01-1.23)  1.09 (0.98-1.22)  1.10 (1.02-1.18) ^b^ | Age; RR |
| Engeland,^38^  1996 | Norwegian parts of the Migrant Study; Norway, 1964-1965 | Cohort, incidence | 1993 (NR) | 11863/707 | Never-smoker  Current  Former  Ever | 139  451  117  568 | Referent  1.10 (0.90-1.30)  0.90 (0.70-1.10)  1.01 (0.83-1.22) ^b^ | Age; RR |
| Veierod,^39^  1997 | Norwegian health screening; Norway, 1977-1983 | Cohort, incidence | 1992 (12.4) | 25708/72 | Never-smoker  Current  Former  Ever | 24  25  20  45 | Referent  0.54 (0.34-0.87) ^b^  0.60 (0.30-1.10)  0.56 (0.38-0.82) ^b^ | Age; RR |
| Cerhan,^40^  1997 | Iowa 65+ Rural Health Study; US, 1981-1982 | Cohort, incidence | 1993 (NR) | 1050/71 | Never-smoker  Current  Former  Ever | 26  15  30  45 | Referent  2.20 (1.20-4.40)  1.20 (0.70-2.10)  1.58 (0.88-2.86) ^b^ | Age; RR |
| Will,^41^  1999 | Cancer Prevention Study I; US, 1959- 1960 | Cohort, incidence | 1972 (NR) | 305065/  2523 | Non-smoker  Current | 1267  1256 | Referent  1.00 (0.92-1.08) | Age; RR |
| Giovannucci,^42^  1999 | Health Professionals Follow-up Study; US, 1986-1994 | Cohort, incidence | 1994  (NR) | 47781/  1369 | Never-smoker  Current  Former  Ever | 580  112  677  789 | Referent  1.04 (0.85-1.27)  1.00 (0.91-1.11) ^b^  1.01 (0.92-1.10) ^b^ | Age, BMI, intakes of calcium, total fat, vitamin E, and lycopene; RR |
| Putnam,^43^  2000 | Iowa Cohort; US, 1986-1989 | Cohort, incidence | 1995  (NR) | 1572/101 | Never-smoker  Current  Former  Ever | 24  16  56  72 | Referent  1.43 (0.80-2.53) ^b^  1.40 (0.90-2.30)  1.41 (0.98-2.03) ^b^ | Age; RR |
| Lund Nilsen,^44^  2000 | Health Survey in Nord-Trøndelag; Norway, 1984-1986 | Cohort, incidence | 1996 (9.3) | 22895/644 | Never-smoker  Current  Former  Ever | 222  153  183  336 | Referent  0.96 (0.78-1.19)  0.98 (0.80-1.19)  0.97 (0.84-1.12) ^b^ | Age; RR |
| Lotufo,^45^  2000 | Physicians' Health Study; US, 1982 | Cohort, incidence | NR (12.5) | 21985/996 | Never-smoker  Current  Former  Ever | 443  96  457  553 | Referent  1.06 (0.85-1.31) ^b^  1.11 (0.98-1.28)  1.10 (0.98-1.23) ^b^ | Age, aspirin assignment, beta-carotene assignment, BMI, height, physical activity, alcohol use; RR |
| Allen,^67^  2004 | Life-Span Study; Japan; 1963-1979 | Cohort, incidence | 1996  (16.9) | 18115/196 | Never-smoker  Ever | 91  101 | Referent  0.80 (0.60-1.07) | Age, calendar period, city of residence,  radiation dose, education level; RR |
| Baglietto,^46^  2006 | Melbourne Collaborative Cohort Study; Australia, 1990-1994 | Cohort, incidence | 2003 (10.3) | 16872/732 | Never-smoker  Current  Former  Ever | 291  76  354  430 | Referent  0.73 (0.57-0.94) ^b^  1.12 (0.96-1.30) ^b^  1.02 (0.88-1.18) ^b^ | None |
| Gonzalez,^47^  2007 | Vitamins and Lifestyle (VITAL); US, 2000-2002 | Cohort, incidence | 2004 (3.3) | 35244/832 | Never-smoker  Current  Former  Ever | 303  62  454  516 | Referent  0.92 (0.70-1.20) ^c^  0.93 (0.78-1.11) ^b^  0.93 (0.80-1.07) ^b^ | Age; HR |
| Park,^48^  2007 | Multiethnic Cohort Study; US, 1993-1996 | Cohort,  incidence | 2002 (NR) | 82483/  4404 | Never-smoker  Current  Former  Ever | 1326  674  2400  3074 | Referent  0.85 (0.77-0.93) ^b^  1.04 (0.98-1.11) ^b^  0.99 (0.93-1.06) ^b^ | None |
| Rohrmann,^49^  2007 | Private Census in Washington County, Maryland; US,1963 | Cohort,  incidence | 1978 (NR) | 26810/147 | Never-smoker  Current  Former  Ever | 34  45  43  88 | Referent  1.00 (0.63-1.59)  1.33 (0.85-2.10)  1.16 (0.84-1.60) ^b^ | Age; RR |
|  | Private Census in Washington County, Maryland; US, 1975 | Cohort,  incidence | 1994 (NR) | 28292/351 | Never-smoker  Current  Former  Ever | 94  85  128  213 | Referent  0.98 (0.73-1.33)  1.04 (0.80-1.36)  1.01 (0.83-1.24) ^b^ | Age; RR |
| Butler,^50^  2009 | Singapore Chinese Health Study; Singapore, 1993- 1998 | Cohort,  incidence | 2006 (10.4) | 27293/250 | Never-smoker  Current  Former  Ever | 108  73  69  142 | Referent  0.88 (0.65-1.19) ^c^  1.06 (0.78-1.44) ^c^  0.96 (0.78-1.20) ^b^ | Age, dialect group, interview year, education, vitamin D, and black tea; HR |
| Watters,^51^  2009 | NIH-AARP Diet and Health Study; US, 1995-1996 | Cohort,  incidence | 2003 (NR) | 283112/ 16640 | Never-smoker  Current  Former  Ever | 5512  1446  9682  11128 | Referent  0.85 (0.80-0.90) ^b^  0.90 (0.87-0.93) ^b^  0.88 (0.83-0.93) ^b^ | None |
| Grundmark,^52^  2011 | Uppsala Longitudinal Study of Adult Men (ULSAM); Sweden, 1970-1974 | Cohort,  incidence | 2003 (26.5) | 2045/208 | Never-smoker  Current  Former  Ever | 69  86  53  139 | Referent  0.60 (0.44-0.83)  0.82 (0.58-1.18)  0.69 (0.51-0.94) ^b^ | None; RR |
| Li,^53^  2011 | Ohsaki Cohort Study; Japan, 1995 | Cohort, incidence | 2003  (NR) | 22458/230 | Non-smoker  Current | 127  94 | Referent  0.64 (0.49-0.83) ^b^ | None |
| Geybels,^54^  2012 | Netherlands Cohort Study; Netherlands, 1986 | Cohort,  incidence | 2003 (NR) | 58279/  3451 | Never-smoker  Current  Former  Ever | 492  1084  1873  2957 | Referent  0.98 (0.82-1.18) ^c^  1.03 (0.87-1.23) ^c^  1.01 (0.89-1.14) ^b^ | Age, duration of smoking, frequency of smoking; HR |
| Karppi,^55^  2012 | Kuopio Ischaemic Heart Disease Risk Factor Study; Finland, 1984-1989 | Cohort,  incidence | 2008 (15) | 997/68 | Non-smoker  Current | 57  11 | Referent  0.49 (0.26-0.93) ^b^ | None;  Another article (Laukkanen et al) from this study reported RR (95% CI) of 0.94 (0.82-1.09) per 10 pack-years (see Supplementary Table 1b below) |
| Shafique,^56^  2012 | Collaborative Study; UK, 1970-1973 | Cohort,  incidence | 2007 (28) | 6017/318 | Never-smoker  Current  Former  Ever | 68  136  114  250 | Referent  0.93 (0.69-1.26) ^c^  1.43 (1.05-1.94) ^c^  1.15 (0.76-1.76) ^b^ | Age, cholesterol, systolic blood pressure, BMI, alcohol intake, tea consumption, smoking status, social class; HR |
| Bae,^57^  2013 | Seoul Male Cancer Cohort Study; South Korea, 1992-1993 | Cohort, incidence | 2008 (NR) | 14450/87 | Never-smoker  Current  Former  Ever | 29  38  19  57 | Referent  0.70 (0.43-1.13)  0.60 (0.34-1.06)  0.66 (0.45-0.95) ^b^ | Age; RR |
| Onitilo,^68^  2013 | Marshfield Clinic; US, 1995-2009 | Cohort, incidence | 2011 (NR) | 33832/ 3432 | Before DM onset Never-smoker Ever After DM onset Never-smoker Ever | 444  1678  389  921 | Referent 1.09 (0.84-1.17) ^c^  Referent 1.20 (1.06-1.35) ^c^ | Date of birth, study time period, residence in the study area, insurance status, BMI, comorbidities; HR |
| Lemogne,^58^  2013 | GAZEL study; France, 1989 | Cohort,  incidence | 2009 (15.2) | 10506/412 | Never-smoker  Current  Former  Ever | NR  NR  NR  NR | Referent  0.71 (0.55-0.91) ^b^  1.09 (0.90-1.32) ^b^  0.89 (0.58-1.35) ^b^ | None |
| Rohrmann,^59^  2013 | European Prospective Investigation into Cancer and Nutrition (EPIC); Europe, 1992-2000 | Cohort,  incidence | 2009 (11.9) | 145112/  4623 | Never-smoker  Current  Former  Ever | 1547  1080  1996  3076 | Referent  0.90 (0.83-0.97) ^c^  0.96 (0.90-1.03) ^c^  0.93 (0.88-0.99) ^b^ | Age, study center, height, weight, education, marital status, vigorous physical activity;  Conducted in Denmark, France, Germany, UK, Greece, Italy, Spain, Norway, The Netherlands, Sweden; HR |
| Sawada,^60^  2014 | Japan Public Health Center-based Prospective Study (JPHC); Japan, 1990-1993 | Cohort,  incidence | 2010 (16) | 48218/913 | Never-smoker  Current  Former  Ever | 257  380  267  647 | Referent  0.79 (0.70-0.90) ^b^  0.84 (0.70-1.00) ^c^  0.81 (0.73-0.89) ^b^ | Age, public health center area, alcohol drinking, BMI, marital status, diabetes, intake of miso soup and Japanese tea; HR |
| Everatt,^61^  2014 | Kaunas–Rotterdam Intervention Study  (KRIS) and Multifactorial Ischemic Heart Disease Prevention Study (MIHDPS); Lithuania, 1972–1974 (KRIS), 1976–1980 (MIHDPS) | Cohort,  incidence | 2008 (NR) | 6976/336 | Never-smoker  Current  Former  Ever | 137  103  96  199 | Referent  0.97 (0.74-1.26) ^c^  0.76 (0.59-1.00) ^c^  0.86 (0.68-1.09) ^b^ | Age, education, alcohol consumption, BMI; HR |
| Ho,^62^  2014 | Reduction by Dutasteride of Prostate Cancer Events (REDUCE) cohort; US, 2003 | Cohort,  incidence | 2009  (NR) | 6420/941 | Never-smoker  Current  Former  Ever | 417  142  382  524 | Referent  1.10 (0.89-1.36)  1.06 (0.91-1.23)  1.07 (0.95-1.21) ^b^ | Age, race, geographic region, PSA, prostate volume, digital rectal examination findings, BMI, treatment arm; OR |
| Perez-Cornago,^6^ 2017 | UK Biobank Cohort Study; UK, 2006-2010 | Cohort,  incidence | 2014  (5.6) | 219335/  4575 | Never-smoker  Current  Former  Ever | 2134  417  1991  2408 | Referent  0.85 (0.77-0.95) ^c^  0.93 (0.88-0.99) ^c^  0.90 (0.83-0.98) ^b^ | Age, region, Townsend deprivation score, ethnicity, lives with a wife or partner, BMI, physical activity, diabetes, enlarged prostate, family history of prostate cancer; HR |
| Jacob,^63^  2018 | Disease Analyzer database (IQVIA); UK,1988-2008 | Cohort,  incidence | 2008  (NR) | 211005/  7744 | Non-smoker  Current | 4632  3112 | Referent  0.71 (0.65-0.76) ^c^ | None; HR |
| Viner,^64^  2019 | Alberta’s Tomorrow Project; Canada, 2001-2008 | Cohort,  incidence | 2017  (12.3) | 10026/401 | Never-smoker  Current  Former  Ever | 158  49  186  235 | Referent  0.70 (0.51-0.98) ^c^  0.85 (0.69-1.06) ^c^  0.80 (0.67-0.96) ^b^ | Age, marital status, education, household income, alcohol consumption, BMI, history of colon cancer screening, history of prostate cancer screening; HR |
| Weber,^65^  2021 | Sax Institute's 45 and Up Study; Australia, 2006-2009 | Cohort,  incidence | 2013  (5.4) | 105143/  4394 | Never-smoker  Current  Former  Ever | 2204  225  1965  2190 | Referent  0.78 (0.68-0.90) ^c^  0.90 (0.84-0.96) ^c^  0.85 (0.74-0.97) ^b^ | Age, remoteness of residence, education, health insurance, country of birth, Socio-Economic Indexes for Areas, alcohol consumption, physical activity, fruit and vegetable consumption, PSA test history, family history of prostate cancer; HR |
| Hippisley-Cox,^66^  2021 | QResearch Database; UK, 1998-2018 | Cohort,  incidence | 2018  (NR) | 844455/  40821 | Never-smoker  Current  Former  Ever | NR  NR  NR  NR | Referent  0.96 (0.93-0.99) ^b^  1.00 (0.98-1.03) ^c^  0.98 (0.94-1.02) ^b^ | Age, BMI, PSA; HR |
| Jochems,^13^  2022 | Five Swedish Cohorts; Sweden, 1974-2016 | Cohort,  incidence | 2016  (28) | 351448/  24731 | Never-smoker  Current  Former  Ever | 10076  7997  6658  14655 | Referent  0.89 (0.86-0.92) ^c^  0.97 (0.94-1.00) ^c^  0.93 (0.85-1.01) ^b^ | Age, geographical region, country of birth, education, and marital status; HR  A pooled analysis of five Swedish cohorts |

**Additional file 1B. Characteristics of the 5 studies not included in the meta-analysis due to lack of information**

| **First author, publication year** | **Study name (or description); country, recruitment period** | **Study design, outcome** | **Last FU (FU^a^, yrs)** | **Total no. men/cases** | **Smoking category*** | **No. cases*** | **RR (95% CI) *** | **Variables the results were adjusted for;**  **Effect measure in the original studies; Other comments** |
| --- | --- | --- | --- | --- | --- | --- | --- | --- |
| Thune,^69^  1994 | Health Screening Program; Norway, 1972-1978 | Cohort, incidence | 1991 (16.3) | 43685/211 | Increment of 10 Cig/day | 211 | 1.08 (0.90-1.30) | Age; RR |
| Tulinius,^70^  1997 | Icelandic Cardiovascular Risk Factor Study; Iceland, 1967-1991 | Cohort, incidence | 1995 (NR) | 11366/524 | Never-smoker  Current  1-14 Cig/day  15-24  25+  Former | NR | NR (NR, NS) | Age |
| Chamie,^71^ 2008 | Northern California Veteran Affairs Health System; US, 1962-1971 | Cohort, incidence | 2006 (NR) | 13144/363 | Smoking history | NR | 0.78 (0.72-0.85) | Age, race, BMI, finasteride use, preoperative PSA, agent orange exposure; OR Smoking history was graded on a scale from 0 to 5 as follows: 0, lifetime nonsmoker; 1, quit >14 years ago; 2, quit >7 years ago; 3, quit >4 years ago; 4, quit in the last year; and 5, current smoker |
| Laukkanen,^72^  2010 | Kuopio Ischemic Heart Disease Risk Factor Study; Finland, 1984-1989 | Cohort, incidence | 2005  (16.7) | 2268/127 | Cigarette pack-years per 10 years | NR | 0.94 (0.82-1.09) | Age, BMI, physical fitness, alcohol consumption; RR |
| Karlsen,^73^  2012 | Danish Diet, Cancer  and Health Study; Denmark, 1993-1997 | Cohort,  incidence | 2000-  2002 (NR) | 20914/129 | Non-smoker  Current  1-10 g/day  11-20  20+ | 81  48  13  22  13 | Referent  1.02 (0.71-1.45) ^b^  1.27 (0.71-2.28) ^b^  1.06 (0.66-1.69) ^b^  0.80 (0.45-1.44) ^b^ | None;  1 g tobacco/cigarette, 4.5 g tobacco/cigar, 3 g tobacco/cheroot, 3 g tobacco/pipe |

FU, follow-up; RR, relative risk; CI, confidence interval; NR, not reported; US, United States; BP, blood pressure; BMI, body mass index; pk-yr, pack-year; yr, year; HR, hazard ratio; UK, United Kingdom; PSA, prostate-specific antigen; DM, diabetes mellitus; NS, non-significant (95% CIs or p-values were not reported, but the authors reported that there was no significant association).

* Data on cigarette smoking.

^a^ The mean or median of follow-up in years.

^b^ RR and 95% CI were calculated using frequency distributions or risk estimates and 95% CIs in subgroups.

^c^ RR and 95% CI were converted from HR and corresponding 95% CI using the formula RR ≈ (1-e ^HR x ln (1-P0)^)/P_0_ (P_0_ refers to the incidence rate of PCa in the control group).
