# Supplementary material for: Association of cigarette smoking habits with the risk of prostate cancer: a systematic review and meta-analysis
Source: BMC Public Health. 2023 Jun 15;23:1150. doi: 10.1186/s12889-023-16085-w (PMC10268475; doi:10.1186/s12889-023-16085-w)
Supplement: Supplementary file 2 — Additional file 2. Results of quality assessment using the Newcastle-Ottawa Scale for cohort studies. [file 12889_2023_16085_MOESM2_ESM.docx]

**Additional file 2. Results of quality assessment using the Newcastle-Ottawa Scale for cohort studies**

| **Study** | **Selection** | | | | **Comparability** | **Outcome** | | | **Quality score** |
| --- | --- | --- | --- | --- | --- | --- | --- | --- | --- |
|  | Representativeness of the exposed cohort | Selection of the non exposed cohort | Ascertainment of exposure | Demonstration that outcome of interest was not present at start of study | Comparability of cohorts on the basis of the design or analysis | Assessment of outcome | Was follow-up long enough for outcomes to occur | Adequacy of follow up of cohorts |  |
| Thompson,^32^  1989 |  | ★ | ★ | ★ | ★★ | ★ | ★ | ★ | 8 |
| Severson,^33^  1989 |  | ★ | ★ | ★ | ★ | ★ | ★ | ★ | 7 |
| Mills,^34^  1989 |  | ★ |  | ★ | ★ | ★ | ★ | ★ | 6 |
| Thune,^69^  1994 | ★ | ★ |  | ★ | ★ | ★ | ★ | ★ | 7 |
| Le Marchand,^35^  1994 | ★ | ★ | ★ | ★ | ★★ | ★ | ★ | ★ | 9 |
| Hiatt,^36^  1994 | ★ | ★ |  | ★ | ★★ | ★ |  | ★ | 7 |
| Adami,^37^  1996 |  | ★ |  | ★ | ★ | ★ | ★ | ★ | 6 |
| Engeland,^38^  1996 | ★ | ★ |  | ★ | ★ | ★ | ★ | ★ | 7 |
| Tulinius,^70^  1997 | ★ | ★ |  | ★ | ★★ | ★ | ★ | ★ | 8 |
| Veierod,^39^  1997 | ★ | ★ |  | ★ | ★ | ★ | ★ | ★ | 7 |
| Cerhan,^40^  1997 | ★ | ★ | ★ | ★ | ★ | ★ | ★ | ★ | 8 |
| Will,^41^  1999 | ★ | ★ |  | ★ | ★ |  | ★ | ★ | 6 |
| Giovannucci,^42^  1999 |  | ★ |  | ★ | ★★ | ★ | ★ | ★ | 7 |
| Putnam,^43^  2000 | ★ | ★ |  | ★ | ★ | ★ | ★ | ★ | 7 |
| Lund Nilsen,^44^  2000 | ★ | ★ |  | ★ | ★ | ★ | ★ | ★ | 7 |
| Lotufo,^45^  2000 |  | ★ |  | ★ | ★★ | ★ | ★ | ★ | 7 |
| Allen,^67^  2004 |  | ★ | ★ | ★ | ★★ | ★ | ★ | ★ | 8 |
| Baglietto,^46^  2006 | ★ | ★ | ★ | ★ |  | ★ | ★ | ★ | 7 |
| Gonzalez,^47^  2007 | ★ | ★ |  | ★ | ★ | ★ |  | ★ | 6 |
| Park,^48^  2007 | ★ | ★ |  | ★ |  | ★ | ★ | ★ | 6 |
| Rohrmann,^49^  2007 | ★ | ★ | ★ | ★ | ★ | ★ | ★ | ★ | 8 |
| Chamie,^71^ 2008 |  | ★ | ★ | ★ | ★★ | ★ | ★ |  | 7 |
| Butler,^50^  2009 |  | ★ | ★ | ★ | ★★ | ★ | ★ | ★ | 8 |
| Watters,^51^  2009 | ★ | ★ |  | ★ | ★★ | ★ | ★ | ★ | 8 |
| Laukkanen,^72^  2010 | ★ | ★ | ★ | ★ | ★★ | ★ | ★ | ★ | 9 |
| Grundmark,^52^  2011 | ★ | ★ | ★ | ★ |  | ★ | ★ | ★ | 7 |
| Li,^53^  2011 | ★ | ★ |  | ★ |  | ★ | ★ | ★ | 6 |
| Geybels,^54^  2012 | ★ | ★ |  | ★ | ★★ | ★ | ★ | ★ | 8 |
| Karlsen,^73^  2012 | ★ | ★ |  | ★ |  | ★ | ★ | ★ | 6 |
| Karppi,^55^  2012 | ★ | ★ | ★ | ★ |  | ★ | ★ | ★ | 7 |
| Shafique,^56^  2012 | ★ | ★ |  | ★ | ★★ | ★ | ★ | ★ | 8 |
| Bae,^57^  2013 | ★ | ★ |  | ★ | ★ | ★ | ★ | ★ | 7 |
| Onitilo,^68^  2013 | ★ | ★ | ★ | ★ | ★★ | ★ | ★ | ★ | 9 |
| Lemogne,^58^  2013 |  | ★ |  | ★ | ★★ | ★ | ★ | ★ | 7 |
| Rohrmann,^59^  2013 | ★ | ★ |  | ★ | ★★ | ★ | ★ | ★ | 8 |
| Sawada,^60^  2014 | ★ | ★ |  | ★ | ★★ | ★ | ★ | ★ | 8 |
| Everatt,^61^  2014 | ★ | ★ | ★ | ★ | ★★ | ★ | ★ |  | 8 |
| Ho,^62^  2014 |  | ★ | ★ | ★ | ★★ | ★ |  | ★ | 7 |
| Perez-Cornago,^6^ 2017 | ★ | ★ |  | ★ | ★★ | ★ | ★ | ★ | 8 |
| Jacob,^63^  2018 | ★ | ★ | ★ | ★ |  | ★ | ★ |  | 6 |
| Viner,^64^  2019 | ★ | ★ |  | ★ | ★★ | ★ | ★ | ★ | 8 |
| Weber,^65^  2021 | ★ | ★ |  | ★ | ★★ | ★ | ★ | ★ | 8 |
| Hippisley-Cox,^66^ 2021 | ★ | ★ | ★ | ★ | ★★ | ★ | ★ | ★ | 9 |
| Jochems,^13^  2022 | ★ | ★ |  | ★ | ★★ | ★ | ★ | ★ | 8 |
