# Supplementary material for: Association of cigarette smoking habits with the risk of prostate cancer: a systematic review and meta-analysis
Source: BMC Public Health. 2023 Jun 15;23:1150. doi: 10.1186/s12889-023-16085-w (PMC10268475; doi:10.1186/s12889-023-16085-w)
Supplement: Supplementary file 3 — Additional file 3. Sensitivity analyses of association between smoking status and risk of prostate cancer. [file 12889_2023_16085_MOESM3_ESM.docx]

**Additional file 3. Sensitivity analyses of association between smoking status and risk of prostate cancer**

| **Variables the results adjusted for** | **No. of studies** | **RR (95% CI)** | **P for**  **overall effect** | **I^2^ statistics** | **P for heterogeneity (Cochran’s Q test)** | **P for publication bias (Egger’s test)** |
| --- | --- | --- | --- | --- | --- | --- |
| **Current smoking** |  |  |  |  |  |  |
| **Reference status** |  |  |  |  |  |  |
| Never smoker | 31 | 0.90 (0.86-0.95) | < 0.001 | 66.7% | < 0.001 | 0.952 |
| Former smoker | 21 | 0.70 (0.65-0.75) | < 0.001 | 81.5% | < 0.001 | 0.725 |
| **Completion year** |  |  |  |  |  |  |
| 1995 or earlier | 7 | 0.79 (0.64-0.98) | 0.033 | 89.8% | < 0.001 | 0.595 |
| After 1995 | 20 | 0.72 (0.66-0.79) | < 0.001 | 91.1% | < 0.001 | 0.045 |
| **World region** |  |  |  |  |  |  |
| North America | 12 | 0.81 (0.72-0.91) | 0.001 | 86.6% | < 0.001 | 0.995 |
| Europe | 9 | 0.69 (0.60-0.80) | < 0.001 | 93.7% | < 0.001 | < 0.001 |
| Asia | 4 | 0.67 (0.60-0.75) | < 0.001 | 0.0% | 0.821 | 0.502 |
| Australia | 2 | 0.63 (0.56-0.70) | < 0.001 | 0.0% | 0.359 | 0.525 |
| **Quality score** |  |  |  |  |  |  |
| ≥ 7 | 21 | 0.71 (0.65-0.79) | < 0.001 | 91.2% | < 0.001 | 0.820 |
| 6 | 6 | 0.81 (0.72-0.92) | 0.001 | 87.5% | < 0.001 | 0.348 |
| **Former smoking** |  |  |  |  |  |  |
| **Completion year** |  |  |  |  |  |  |
| 1995 or earlier | 10 | 1.04 (0.98-1.11) | 0.205 | 6.1% | 0.386 | 0.759 |
| After 1995 | 21 | 0.97 (0.94-1.01) | 0.107 | 69.1% | < 0.001 | 0.991 |
| **World region** |  |  |  |  |  |  |
| North America | 13 | 1.01 (0.95-1.09) | 0.681 | 65.2% | < 0.001 | 0.080 |
| Europe | 13 | 0.98 (0.94-1.02) | 0.298 | 50.8% | 0.018 | 0.721 |
| Asia | 3 | 0.87 (0.75-1.01) | 0.060 | 40.9% | 0.184 | 0.637 |
| Australia | 2 | 0.99 (0.80-1.23) | 0.948 | 85.1% | 0.010 | 0.290 |
| **Quality score** |  |  |  |  |  |  |
| ≥ 7 | 27 | 0.97 (0.94-1.01) | 0.104 | 60.3% | < 0.001 | 0.447 |
| 6 | 4 | 1.05 (0.99-1.10) | 0.084 | 14.0% | 0.322 | 0.725 |
| **Ever smoking** |  |  |  |  |  |  |
| **Completion year** |  |  |  |  |  |  |
| 1995 or earlier | 10 | 1.05 (1.00-1.10) | 0.046 | 49.7% | 0.030 | 0.581 |
| After 1995 | 23 | 0.95 (0.91-0.99) | 0.011 | 66.7% | < 0.001 | 0.212 |
| **World region** |  |  |  |  |  |  |
| North America | 14 | 1.02 (0.96-1.09) | 0.541 | 68.4% | < 0.001 | 0.150 |
| Europe | 13 | 0.95 (0.90-1.01) | 0.075 | 63.2% | 0.001 | 0.070 |
| Asia | 4 | 0.82 (0.76-0.89) | < 0.001 | 12.8% | 0.329 | 0.837 |
| Australia | 2 | 0.93 (0.78-1.11) | 0.419 | 68.8% | 0.073 | 0.363 |
| **Quality score** |  |  |  |  |  |  |
| ≥ 7 | 29 | 0.96 (0.92-1.00) | 0.047 | 66.0% | < 0.001 | 0.915 |
| 6 | 4 | 1.02 (0.94-1.10) | 0.691 | 53.7% | 0.091 | 0.578 |

RR, relative risk; CI, confidence interval; PSA, prostate-specific antigen
